# Effects of Shenfu injection on inflammatory factors and immune function in children with *Mycoplasma pneumoniae*: A protocol for a double-blind, randomized controlled trial

**DOI:** 10.1097/MD.0000000000027585

**Published:** 2021-10-22

**Authors:** Honglian Pei, Youfeng Ma, Lin Wang, Liping Wang, Li Xu, Rong Wang

**Affiliations:** a Department of Pediatrics, Shenzhen Samii International Medical Center, Shenzhen, Shenzhen Province, China; b Department of Clinical Lab, Weinan Maternal and Child Health Hospital, Shaanxi, Weinan Province, China.

**Keywords:** azithromycin, children, *Mycoplasma pneumoniae*, protocol, randomized controlled trial, Shenfu injection

## Abstract

**Background:**

*Mycoplasma pneumoniae* (MP) is a common infectious respiratory disease in pediatrics, and macrolide antibiotics are the optimal treatment option. In recent years, there is a significant increase in the resistance of this pathogen to macrolide antibiotics, which makes the clinical treatment of this disease increasingly complex. Shenfu injection (SFI), a herbal extract injection, has advantages of improving immune function, reducing inflammatory reaction, improving curative effect and shortening the course of disease in the treatment of pediatric MP. However, there is a lack of rigorous clinical studies to evaluate the effects of SFI on inflammatory factors and immune function in children with MP.

**Methods:**

This study is a prospective, randomized, double-blind, placebo-controlled clinical trial protocol. The objective of this study is to evaluate the effect of SFI on inflammatory factors and immune function in children with MP. Patients meeting the inclusion criteria were randomized in a ratio of 1:1 to either the treatment group (azithromycin + 100 mL 5% glucose injection + 50 mL SFI) or the control group (azithromycin + 150 mL 5% glucose injection). Patients in both groups received the standard treatment for 7 days. The levels of inflammatory factor indexes (C-reactive protein, interleukin-6, interleukin-10, tumor necrosis factor-α) and immune function indexes (immunoglobulin G, immunoglobulin A, immunoglobulin M) in both groups were measured at the beginning of treatment, on the 3rd day of treatment and at the end of treatment. Besides, the time of improvement in clinical symptoms (duration of cough, time of disappearance of lung rales, time of fever reduction, and time of disappearance of lung X-ray infiltrative shadow) and adverse effects in both groups were recorded. Finally, the data were statistically analyzed by SPSS 20.0 software.

**Discussion:**

In this study, an evaluation was conducted on the effects of SFI on inflammatory factors and immune function in pediatric MP. The results of this experiment will provide a clinical basis for the adjuvant treatment of pediatric MP with SFI.

**Trial registration:**

OSF Registration number:

## 1 Introduction

*Mycoplasma pneumoniae* (MP) is a common lower respiratory tract infection disease in children, accounting for 10%∼20% of the incidence of pediatric pneumonia, which can occur throughout the year and exert severe impacts on the life and health of children.^[1]^ The clinical manifestations of MP mainly include fever, paroxysmal irritating cough, wheezing, dyspnea, and so forth. In severe cases, such extra-pulmonary symptoms as the digestive system and nervous system may even appear.^[2]^ MP can not only induce pharyngitis, tonsillitis, and other respiratory tract infections, but also contribute to meningitis, hepatitis, myocarditis, and so forth. In severe cases, it can also lead to the death of children.^[3]^

In western medicine, macrolide antibiotics are favored in the clinical treatment of MP. The clinical effectiveness of azithromycin is even definitely confirmed. However, due to its side effects, such as gastrointestinal reactions and hepatotoxicity, this agent cannot be used for a long time, with a long course of treatment and slow improvement of symptoms.^[4,5]^ It is therefore necessary to seek new alternative therapies.

Chinese herbal injections are made from ingredients that are extracted from Chinese herbs through modern technology, and they are characterized by rapid action and high bioavailability.^[6]^ Shenfu injection (SFI) is a common clinical Chinese medicine injection, and its main ingredients are aconitum alkaloids and ginsenosides, which can promote Yang and support the essence, reduce blood viscosity, improve the patient's ischemic, and hypoxic tolerance, improve the symptoms of spleen deficiency and qi deficiency, effectively enhance the patient's heart function, and relieve the smooth muscle spasm of bronchus, so that the damaged lung tissue cells can be protected and the immune function of the body can be enhanced.^[7,8]^

SFI has been used in the treatment of pediatric MP for quite a few years. It features the improvement in efficiency, the shortening of the course of the disease, and the decrease in the side effects of azithromycin.^[9]^ However, there is currently no rigorous randomized controlled trial to explore the efficacy of SFI combined with azithromycin in the treatment of MP in children. Therefore, an evaluation will be conducted on the efficacy and safety of SFI in combination with azithromycin in the treatment of MP in children via this randomized controlled trial.

## 2 Materials and methods

### 2.1 Study design

This is a prospective, randomized, double-blind, placebo-controlled study protocol to investigate the effects of SFI on inflammatory factors and immune function in children with MP. The protocol conforms to the Standard Protocol Items: Recommendations for Interventional Trials 2013 Statement,^[10]^ and the results were reported according to the Consolidated Standards of Reporting Trials Statement extension for trials.^[11]^

### 2.2 Ethics and registration

The study protocol was formulated in accordance with the Declaration of Helsinki and approved by our Clinical Research Ethics Committee. This experiment has been registered in the open science framework (registration number:). Prior to the start of the experiment, the family members of these patients were informed in detail with respect to the purpose, methods and potential risks of this experiment through a face-to-face meeting. A written informed consent form was provided by the family members of these patients after obtaining their consent. These patients or their family members can choose at any time whether to continue with the trial.

### 2.3 Patients

Inclusion criteria:

(1) Diagnosed on the basis of such clinical symptoms as irritating cough and chest imaging, and a positive IgM test for serum MP specific antibodies;
(2) Aging ≥1 year and ≤12 years;
(3) Diagnosed within 48 hours; and
(4) The children were able to actively cooperate in completing the treatment, and the child's family members would provide the informed consent and sign a written informed consent form.

Exclusion criteria:

(1) Patients in critical condition or with other respiratory infections, such as acute upper respiratory tract infections;
(2) Chest X-ray showing lung tumors, tuberculosis;
(3) Concomitant with acute infectious diseases, such as measles and influenza;
(4) Concomitant with serious primary diseases of the heart, liver, kidney, digestive, and hematopoietic systems;
(5) Patients who have participated or are participating in clinical trials of other drugs within the last 3 months; and
(6) Allergy to drugs used in this study.

Rejection and shedding criteria:

(1) Patients who would not complete the treatment according to the protocol after the start of the study;
(2) Patients who would have poor adherence and compliance with medical advice;
(3) Patients who would fail to complete the treatment and ask to quit during the study;
(4) Patients who would suffer from serious complications or changes in their condition during the course of the study that prevented them from continuing treatment; and
(5) Patients who would receive other combined treatments that were not within the prescribed range and would exert a greater impact on the outcome of the trial.

For those cases excluded or dislodged from this study, we would make every endeavor to complete the last test, so that the efficacy and safety could be analyzed and appropriate treatment measures could be performed. Reasons for all excluded or dislodged cases were entered on the case report form.

### 2.4 Sample size

The sample size estimation was based on the mean and standard deviation of the treated children's C-reactive protein scores with reference to the pretest results of 10.87 ± 2.63 for the treatment group and 13.14 ± 2.91 for the control group. α = 0.025, one-sided test, and β = 0.10 were established. According to the results calculated by PASS 15.0 software (NCSS, LLC. Kaysville, UT, USA), 33 patients per group were required. Due to an estimated dropout rate of 20%, 42 patients were eventually included in each group.

### 2.5 Randomization and blinding

**Figure 1:**
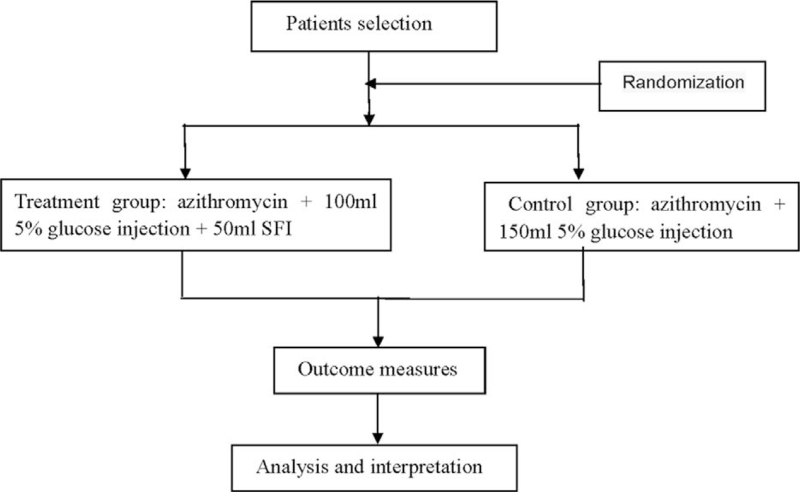
Flow diagram of the trial. SFI = Shenfu injection.

Those patients who meet the criteria were randomized into the treatment and control groups in a ratio of 1:1 (Fig. 1). Random numbers were generated by SPSS 20.0 software (version 20.0; SPSS Inc., Chicago, Illinois, United States) and were placed in sealed and opaque envelopes with numbers written on them. The envelopes were randomly distributed to the family members of these children and the randomization were completed based on the random numbers. Throughout the study, these children, their family members, and the principal investigator were unaware of the results of the randomization. The final statistical analysis of the study data was completed by a statistician who was not involved in the randomization.

### 2.6 Intervention measures

Children in both groups received the conventional treatment methods, such as cough and wheeze suppression, antipyretic, expectorant, and rehydration upon admission. The treatment details were recorded in detail in the case report form. The control group: azithromycin (Guorui Pharmaceutical Co., Ltd., GMP H20010189, China) 10 mg/kg + 150 mL 5% glucose injection, intravenous drip, once daily for 7 days. The treatment group: azithromycin 10 mg/kg + 100 mL 5% glucose injection + 50 mL SFI (Sanjiu Medical & Pharmaceutical Co., LTD. Z20043117, China), intravenous drip, once daily for 7 days. For the fact that SFI is a colored transparent liquid, nurses used the disposable light-proof infusion cover to effectively block the test drug and 5% glucose injection in order to reduce bias. In this study, relevant doctors, evaluators, patients and statistical experts were blinded, except for relevant nurses.

### 2.7 Observation indexes

(1) The main outcome: the duration of hospital stay; and
(2) The secondary outcomes include:
  i) time of improvement in clinical symptoms, including duration of cough, time of disappearance of lung rales, time of fever reduction, and time of disappearance of lung X-ray infiltrates;
  ii) inflammatory factor indexes and immune function indexes: the cubital venous blood were collected from these children on an empty stomach before treatment, on the 3rd day of the treatment and in the early morning after the end of treatment. After the process of these blood samples, C-reactive protein, interleukin-6, interleukin-10, and tumor necrosis factor-α were detected by enzyme-linked immunosorbent assay. Immunoglobulin A, immunoglobulin M, and immunoglobulin G were detected by flow cytometry (Beckman, EPICS XL, US); and
  iii) adverse reactions: any symptoms of discomfort during this study, including gastrointestinal reactions, rash, dizziness, etc.

### 2.8 Safety evaluation

These patients’ blood routine, urine routine, liver function (glutamic aminotransferase and glutamic aminotransferase) and renal function (urea nitrogen and blood creatinine) were subjected to an examination at baseline and after the end of treatment, with the aim of assessing the safety of the treatment. Adverse events occurring during the treatment were also recorded with a detailed description, including the time of occurrence, duration of symptoms, severity of symptoms, management measures, time of resolution of adverse effects, and causality classification. The investigator recorded and managed all adverse events, whether or not they were relevant to the treatment drug in this study.

### 2.9 Data collection and management

In the entire trial process, the clinical trial quality management specifications were strictly implemented. All study data were recorded completely, truthfully, clearly, and objectively in the case report form, and the trial data were entered into the computer after being completely recorded and stored after locking the data. In order to ensure data accuracy, 2 data entry clerks entered and proofread the data independently. The database was locked by the data manager after data verification and may not be changed again.

### 2.10 Statistical analysis

The collected data were statistically analyzed by SPSS 20.0 software. Count data were analyzed by chi-square test; measurement data were expressed as mean ± standard deviation ($\bar{x}$ ± S). Besides, the normal distribution was analyzed by independent samples *t* test, and the skewed distribution was analyzed by Mann–Whitney *U* test. *P* < .05 was considered a statistically significant difference.

### 2.11 Monitoring plan

This study has been approved by the Ethics Committee of this hospital and is under its supervision. Regular monitoring shall be maintained during this study, in an attempt to ensure that the patient enrollment meets inclusion and exclusion criteria, the study process complies with ethical norms, and the application of study data is regulated and confidential. The trial operations were carried out in strict accordance with the protocol and any changes to the study plan would be approved by the Ethics Committee.

## 3 Discussion

Due to the irregular treatment strategies and the abuse of macrolides in the clinic, as well as the transmission of drug-resistant strains, some ribosomal nucleotide sites have been mutated, which results in increased resistance of mycoplasma to azithromycin.^[12]^ The application of azithromycin may also induce repolarization of the body's ventricles, which may cause arrhythmias and such risks as tachycardia.^[9]^ The combination of Chinese and Western medicine in the treatment of MP in children has become a common practice in China and has developed into a comprehensive treatment model.^[13,14]^

It is becoming a research hotspot for the treatment of MP in children with Chinese medicine injections.^[15]^ The application of SFI combined with azithromycin in the treatment group of this study for pediatric MP will be expected to compensate for the shortcomings of azithromycin alone for this disease, achieve improved immune function, reduce the inflammatory response, and thus increase the efficacy and shorten the course of the disease. Therefore, this combined treatment strategy is worthy of future clinical promotion. For the fact that this study is a single-center study and the geographical nature of the included population may have some influence on the results, more randomized controlled trials with multiple centers and large sample sizes shall be conducted to validate these conclusions in this study.

## Author contributions

**Conceptualization:** Honglian Pei, Rong Wang.

**Data curation:** Youfeng Ma.

**Formal analysis:** Youfeng Ma.

**Funding acquisition:** Rong Wang.

**Investigation:** Youfeng Ma.

**Methodology:** Lin Wang.

**Project administration:** Rong Wang.

**Resources:** Lin Wang.

**Software:** Lin Wang.

**Supervision:** Rong Wang.

**Validation:** Liping Wang, Li Xu.

**Visualization:** Liping Wang, Li Xu.

**Writing – original draft:** Honglian Pei, Rong Wang.

**Writing – review & editing:** Honglian Pei, Rong Wang.

## References

[1] Li L. Progress in the treatment of mycoplasma pneumonia in children. China Contin Med Educ 2020;12:132–3.

[2] Zhu Z Zhang T Guo W Ling Y Tian J Xu Y. Clinical characteristics of refractory *Mycoplasma pneumoniae* pneumonia in children treated with glucocorticoid pulse therapy. BMC Infect Dis 2021;21:126. 33509121 10.1186/s12879-021-05830-4 PMC7844890

[3] Biondi E Mcculloh R Alverson B Klein A Ralston S. Treatment of mycoplasma pneumonia: a systematic review. Pediatrics 2014;133:1081. 24864174 10.1542/peds.2013-3729

[4] Jiang H Zheng X Zeng Y Ma Y Peng W. Randomized controlled trial and Meta-analysis of the therapeutic effect of Maxing Shigan decoction combined with azithromycin on *Mycoplasma pneumoniae* pneumonia in children. Chin Pediatrics Integr Tradit West Med 2021;13:131–7.

[5] Parnham MJ Erakovic Haber V Giamarellos-Bourboulis EJ Perletti G Verleden GM Vos R. Azithromycin: mechanisms of action and their relevance for clinical applications. Pharmacol Ther 2014;143:225–45. 24631273 10.1016/j.pharmthera.2014.03.003

[6] Li J-P Liu Y Guo J-M. A comprehensive strategy to evaluate compatible stability of chinese medicine injection and infusion solutions based on chemical analysis and bioactivity assay. Front Pharmacol 2017;8:833–1833. 29187820 10.3389/fphar.2017.00833 PMC5694823

[7] Huang J Wang Y Huang S. A critical overview of systematic reviews of Shenfu injection for heart failure. Cardiovasc Ther 2021;2021:8816590. 33777184 10.1155/2021/8816590 PMC7960068

[8] Zhang N Liu J Qiu Z Ye Y Zhang J Lou T. Shenfu injection for improving cellular immunity and clinical outcome in patients with sepsis or septic shock. Am J Emerg Med 2017;35:01–6. 10.1016/j.ajem.2016.09.008 28029485

[9] Feng J. Effects of azithromycin combined with Shenfu injection on serum IL-2, IL-22 and humoral immune function in children patients with mycoplasmal pneumonia. Drug Eval 2021;18:81–3.

[10] Chan AW Tetzlaff JM Gøtzsche PC. SPIRIT 2013 explanation and elaboration: guidance for protocols of clinical trials. BMJ (Clin Res ed) 2013;346:e7586. 10.1136/bmj.e7586 PMC3541470 23303884

[11] Moher D Hopewell S Schulz KF. CONSORT 2010 explanation and elaboration: updated guidelines for reporting parallel group randomised trials. BMJ (Clin Res ed) 2010;340:c869. 10.1136/bmj.c869 PMC2844943 20332511

[12] Sun J Yan J Qu J. Clinical effect of aerosol inhalation of budesonide and terbutaline combined with sequential therapy of azithromycin in treating mycoplasma pneumonia in children and the impact on serum levels of inflammatory cytokines, sB7-H3 and G-CSF. Pract J Card Cereb Pneum Vasc Dis 2019;27:89–93.

[13] Zhang GM Huang ZY Sun R Ye SL Feng Q. Xiao’er Xiaoji Zhike oral liquid combined with azithromycin for *Mycoplasma pneumoniae* pneumonia in children: a systematic review and meta-analysis. Evid Based Complement Alternat Med 2020;2020:01–13. 10.1155/2020/9740841 PMC7387976 32765636

[14] Jin L Chen P Xu Y. Effects of four types of Chinese medicines as concomitant drugs with azithromycin for the treatment of mycoplasma pneumonia in children in China: a network meta-analysis. Rev Assoc Med Bras (1992) 2021;67:395–9. 34468604 10.1590/1806-9282.20200808

[15] Duan X Wang K Wu J. Comparative efficacy of Chinese herbal injections combined with azithromycin for mycoplasma pneumonia in children: a Bayesian network meta-analysis of randomized controlled trials. J Clin Pharm Ther 2019;44:675–84. 31119782 10.1111/jcpt.12855 PMC6852301

